# ERβ inhibits cyclin dependent kinases 1 and 7 in triple negative breast cancer

**DOI:** 10.18632/oncotarget.21787

**Published:** 2017-10-11

**Authors:** Jordan M. Reese, Elizabeth S. Bruinsma, David G. Monroe, Vivian Negron, Vera J. Suman, James N. Ingle, Matthew P. Goetz, John R. Hawse

**Affiliations:** ^1^ Department of Molecular Pharmacology and Experimental Therapeutics, Mayo Clinic, Rochester, MN, USA; ^2^ Department of Biochemistry and Molecular Biology, Mayo Clinic, Rochester, MN, USA; ^3^ Department of Pathology, Mayo Clinic, Rochester, MN, USA; ^4^ Department of Biomedical Statistics and Informatics, Mayo Clinic, Rochester, MN, USA; ^5^ Department of Oncology, Mayo Clinic, Rochester, MN, USA

**Keywords:** TNBC, ERβ, cell cycle, CDK, estrogen

## Abstract

Triple negative breast cancer (TNBC), which comprises approximately 15% of all primary breast cancer diagnoses, lacks estrogen receptor alpha, progesterone receptor and human epidermal growth factor receptor 2 expression. However, we, and others, have demonstrated that approximately 30% of TNBCs express estrogen receptor beta (ERβ), a nuclear hormone receptor and potential drug target. Treatment of ERβ expressing MDA-MB-231 cells with estrogen or the ERβ selective agonist, LY500307, was shown to result in suppression of cell proliferation. This inhibitory effect was due to blockade of cell cycle progression. *In vivo*, estrogen treatment significantly repressed the growth of ERβ expressing MDA-MB-231 cell line xenografts. Gene expression studies and ingenuity pathway analysis identified a network of ERβ down-regulated genes involved in cell cycle progression including CDK1, cyclin B and cyclin H. siRNA mediated knockdown or drug inhibition of CDK1 and CDK7 in TNBC cells resulted in substantial decreases in proliferation regardless of ERβ expression. These data suggest that the tumor suppressive effects of ERβ in TNBC result from inhibition of cell cycle progression, effects that are in part mediated by suppression of CDK1/7. Furthermore, these data indicate that blockade of CDK1/7 activity in TNBC may be of therapeutic benefit, an area of study that has yet to be explored.

## INTRODUCTION

Triple negative breast cancer (TNBC) accounts for approximately 15% of all breast cancers diagnosed [1, 2] and is defined by the absence of estrogen receptor alpha (ERα), progesterone receptor (PR), and human epidermal growth factor receptor 2 (HER2) amplification [1, 2]. TNBC typically occurs in young, pre-menopausal women and is more prevalent in women of African-American decent [3]. Women diagnosed with TNBC usually present with larger tumors of higher grade that have spread to the lymph nodes [4]. Despite current treatment strategies which include surgery, chemotherapy, and radiation therapy, patients with TN disease are faced with a poor prognosis [5]. In fact, 34% of patients with newly diagnosed TNBC will develop recurrent disease within five years of diagnosis following aggressive chemotherapy treatment [5]. Therefore, a better understanding of the molecular pathways responsible for driving TNBC development and progression, and the concurrent identification of novel therapeutic drug targets, are vital steps to combatting this breast cancer subtype.

We have previously demonstrated that a second form of the estrogen receptor, ERβ, is expressed in approximately 30% of TNBCs [6] and have shown that proliferation of ERβ+ TNBC cell lines is significantly inhibited following estrogen treatment [6-8]. A recent meta-analysis of ERβ expression in breast cancer showed significant associations with increased disease-free survival (DFS) and overall survival (OS) in ERα-negative patients [9]. These data support the notion that drugs designed to specifically activate ERβ may elicit therapeutic benefit in the portion of TNBC patients with ERβ+ disease.

Past studies have suggested that one of the mechanisms by which ERβ functions as a tumor suppressor in breast cancer is through alterations in cell cycle progression [8, 10, 11]. Cell division is a well-orchestrated process that requires coordinated expression of key factors during specific points in the cell cycle in order to be successful. These factors include cyclin-dependent kinases (CDKs) which are heterodimeric serine/threonine kinases that depend on cyclins, their binding partners, for catalytic activity [12, 13]. In humans, there are 21 CDK genes [14] which are known to be positive regulators of cell proliferation, gene transcription and mRNA processing [15, 16].

Abnormal activation of CDKs results in increased proliferation of cancer cells and genomic instability [17]. For these reasons, inhibition of CDK activity has received significant attention as a therapeutic approach to treat multiple forms of cancer. In breast cancer, CDK4 and CDK6 are among the most well studied CDKs. CDK4 and 6 interact with cyclin D to drive cell cycle progression from G0 to early G1 phase [18]. Palbociclib, an inhibitor of CDK4 and CDK6, in combination with endocrine therapy resulted in improved progression-free survival (PFS) in ERα+/Her2 negative advanced breast cancer [19-21]. Unfortunately, palbociclib does not inhibit proliferation in TNBC cell lines [19, 22] although a recent study has demonstrated that blockade of CDK4/6 suppresses TNBC metastases [23].

In the current study, we demonstrate that treatment of ERβ expressing TNBC cells with estrogen, or the ERβ selective agonist, LY500307, results in suppression of proliferation through blockade of cell cycle progression. Furthermore, activation of ERβ was shown to significantly inhibit the growth of MDA-MB-231 cell line xenografts *in vivo*. From a mechanistic standpoint, ligand-mediated activation of ERβ results in the suppression of a network of genes involved in cell cycle progression including CDK1, CDK7, cyclin B and cyclin H. siRNA-mediated depletion, or drug inhibition of CDK1 and CDK7 resulted in substantial decreases in TNBC cell proliferation, effects that were independent of ERβ expression. Taken together, these data suggest that the tumor suppressive effects of ERβ in TNBC are in part mediated by inhibition of genes involved in cell cycle progression and provide further support for the development of CDK1 and CDK7 specific inhibitors for the treatment of TNBC.

## RESULTS

### The selective ERβ agonist, LY500307, inhibits TNBC cellular proliferation

**Figure 1:**
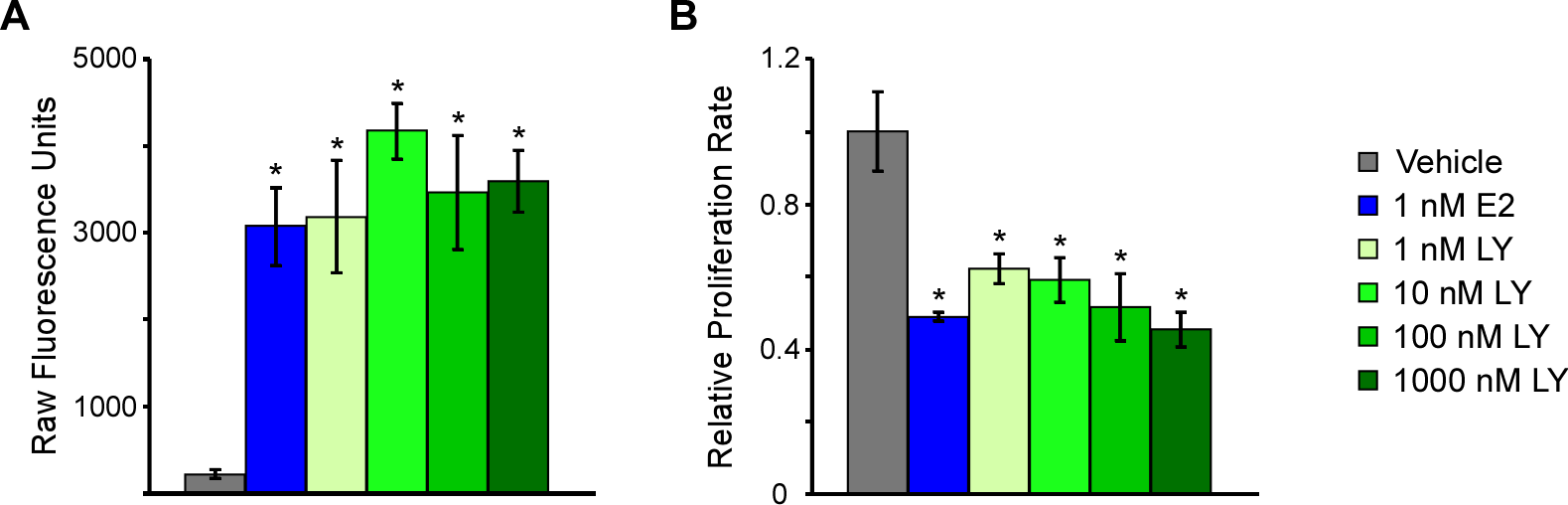
Estrogen, and the ERβ-agonist, LY500307, activate ERβ transcriptional activity and decrease cell proliferation in TNBC cells **A.** A luciferase reporter construct containing an estrogen response element was transiently transfected into ERβ expressing MDA-MB-231 cells and subsequently treated as indicated for 24 hours prior to assessing luciferase activity. **B.** Crystal violet assays indicating the effects of increasing concentrations of LY500307, an ERβ-selective agonist, on MDA-MB-231-ERβ cell proliferation rates relative to vehicle control or E2 treatment. Treatments were performed in the presence of doxycycline for five days. * Denotes significance at the *P ≤* 0.05 level compared with vehicle control.

We, and others, have shown that approximately 30% of all TNBCs express ERβ [6, 24] and that estrogen treatment of ERβ expressing TNBC cell lines results in decreased proliferation rates [6, 7]. Using a doxycycline (Dox)-inducible ERβ expressing MDA-MB-231 cell line generated in our laboratory, we have confirmed that ligand mediated transcriptional activation of ERβ with either estrogen or multiple doses of the highly selective ERβ agonist, LY500307, (Figure 1A) results in significant inhibition of TNBC cell proliferation following five days of treatment (Figure 1B). All concentrations of LY500307 used in this study resulted in significant repression of cell proliferation relative to vehicle control treated cells, effects that were nearly identical to that of 1 nM estrogen treatment.

### ERβ activation does not induce apoptosis

**Figure 2:**
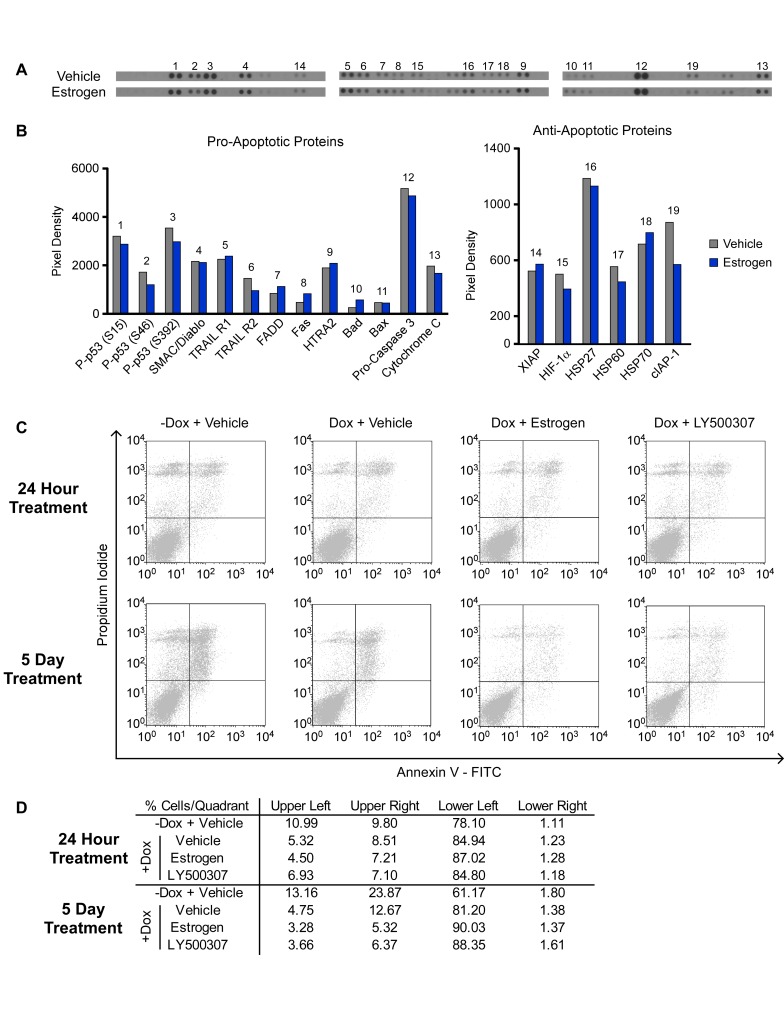
Effects of ERβ on Apoptosis of TNBC cells **A.** and **B.** A protein-based apoptosis array was used to assess the effects of 1 nM E2 treatment (24 hours) of ERβ expressing MDA-MB-231 cells on the expression levels of multiple pro- and anti-apoptotic proteins. **C.** and **D.** Annexin V staining of MDA-MB-231-ERβ cells following 24 hours and 5 days of treatment with vehicle, 1 nM estrogen or 10 nM LY500307 as determined by flow cytometry.

Given the observed decreases in TNBC cell proliferation following estrogen and ERβ selective agonist treatment, we next sought to determine if these compounds induced programmed cell death. Using an antibody based array, we analyzed the impact of 24 hours of estrogen treatment on the expression levels of 35 different apoptosis-related proteins in MDA-MB-231-ERβ cells. The expression levels of classic pro-apoptotic proteins such as cytochrome C, pro-caspase 3, and FADD did not significantly change with treatment, nor did a number of anti-apoptotic proteins such as XIAP, HIF-1α and cIAP (Figure 2A and 2B). Furthermore, Annexin V staining followed by flow cytometry did not indicate any induction of apoptosis following estrogen or LY500307 treatment for 24 hours or 5 days (Figure 2C and 2D). Instead, a decrease in the percentage of apoptotic cells (as indicated by the number of cells in the upper-right quadrant of the scatterplot) was observed following induction of ERβ expression, an effect that was further magnified in the setting of ligand treatment (Figure 2C and 2D). These data indicate that ERβ-mediated decreases in TNBC cell proliferation are not due to the induction of programed cell death.

### Ligand mediated activation of ERβ induces cell cycle arrest

**Figure 3:**
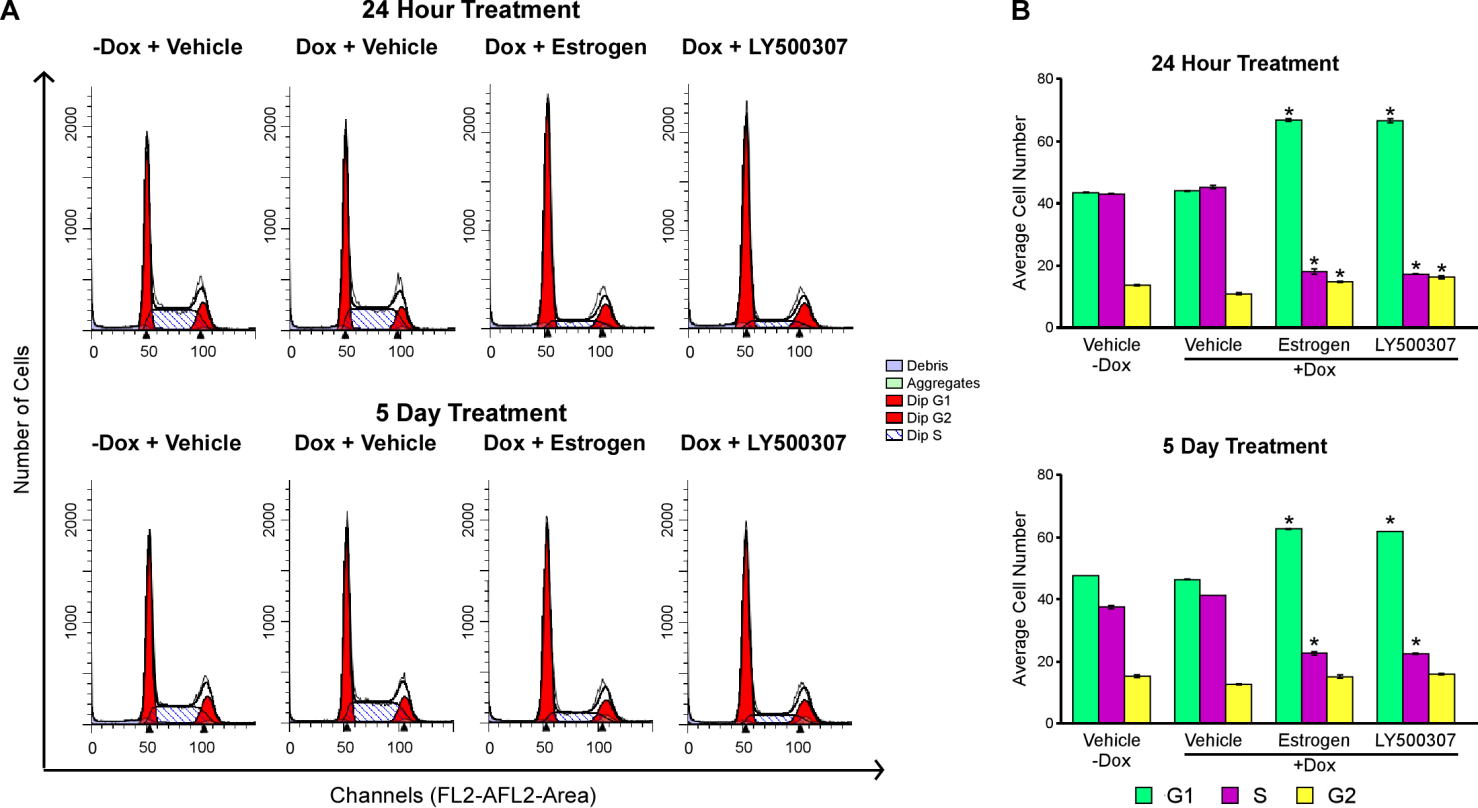
Ligand mediated activation of ERβ suppresses cell cycle progression Flow cytometry analysis of MDA-MB-231-ERβ cells following doxycycline induction of ERβ, as well as ligand mediated activation of ERβ with 1 nM E2 and 10 nM LY500307 for 24 hours or 5 days. **A.** Raw peaks and **B.** quantitation of cells in each phase of the cell cycle following indicated treatments. * Denotes significance at the P ≤ 0.05 level compared with vehicle control.

Based on the above findings, we next assessed the impact of estrogen and LY500307 on cell cycle progression. Following 24 hours or 5 days of treatment with estrogen or LY500307, propidium iodide (PI) staining followed by flow cytometry analysis revealed a statistically significant accumulation of cells in the G1 phase of the cell cycle compared to vehicle-treated controls (Figure 3A and 3B). These data indicate that the tumor suppressive effects of ERβ in TNBC cells primarily result from the induction of cell cycle arrest.

### Estrogen suppresses ERβ positive TNBC tumor growth *in vivo*

**Figure 4:**
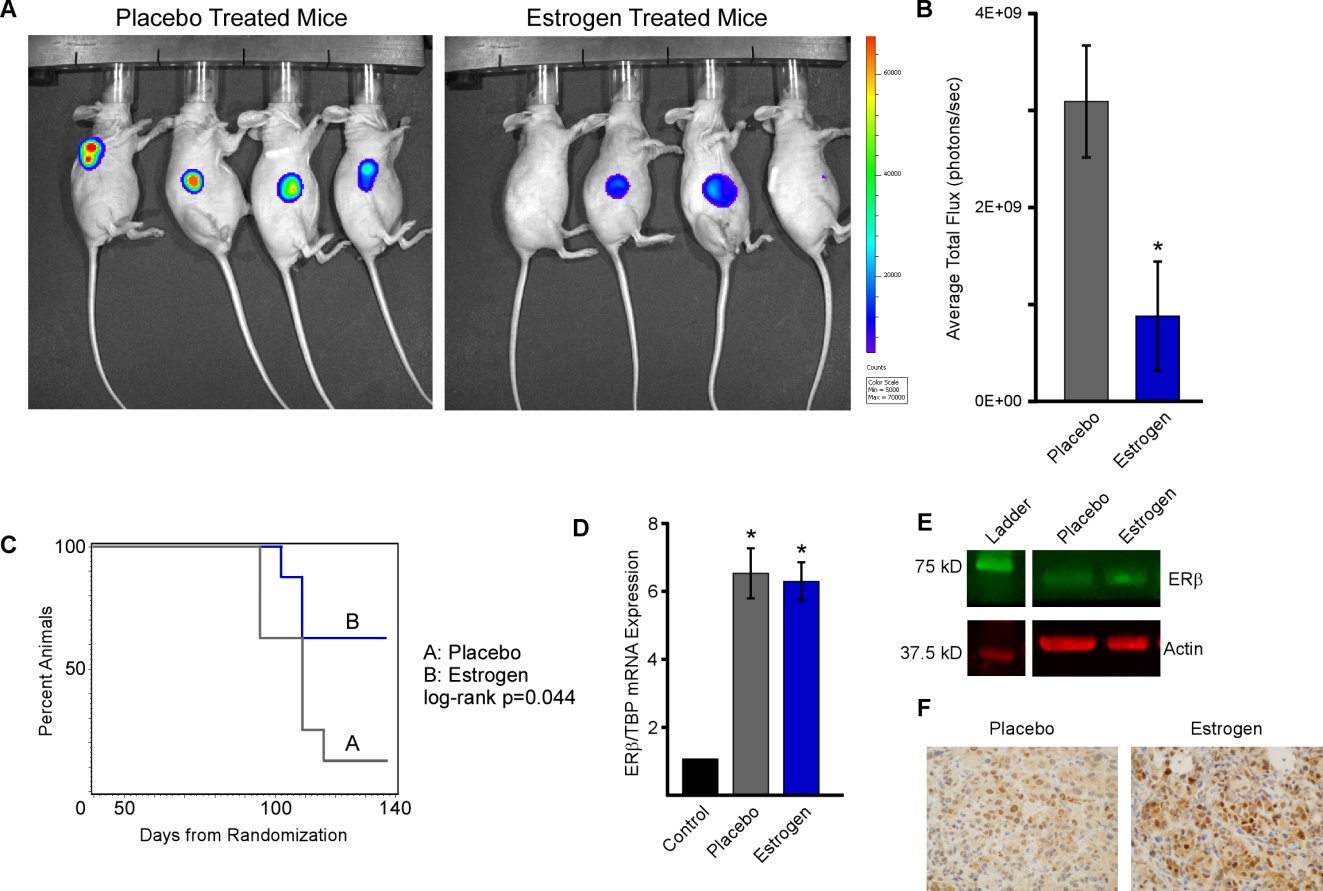
Estrogen inhibits MDA-MB-231-ERβ cell line xenograft growth Ovariectomized athymic nude mice harboring MDA-MB-231-ERβ-Luc cell line xenografts were randomized to placebo control or E2 treatment groups (*n* = 8 animals per group). **A.** Tumor progression was monitored at 8 weeks *via* IVIS2000 xenogen imaging and **B.** average total flux was quantified. **C.** Time to tumor doubling was calculated following weakly tumor measurements and a log-rank test was used to assess difference between treatment groups. **D.** The level of ERβ mRNA expression was determined in residual tumors isolated from placebo and E2 treated mice at the time of sacrifice by RT-PCR and was normalized to No Dox/Veh control treated cells. **E.** Western blotting and **F.** immunohistochemistry of tumor lysates and FFPE tumor sections respectively indicating ERβ protein expression levels in residual tumors isolated from placebo and E2 treated mice. * Denotes significance at the *P* ≤ 0.05 level compared with vehicle control.

Given these *in vitro* findings, we next examined the effect of ERβ activation on MDA-MB-231 tumor growth *in vivo*. One million MDA-MB-231-ERβ-Luc cells were implanted subcutaneously into the right flank of 6-8 week old ovariectomized female nude mice and tumors were allowed to form and progress to a tumor volume of approximately 100 mm^3^. Following tumor establishment, animals were placed on doxycycline-containing chow and randomized to placebo or estrogen pellets. Tumor volume was determined weekly using calipers and luciferase readouts were captured using the IVIS2000 xenogen machine after 8 weeks of treatment (Figure 4A and 4B). Animal weights were also measured weekly as a means of monitoring toxicities and no changes were observed during the course of the study. Estrogen treatment resulted in suppression of tumor progression as indicated by a significantly increased time to tumor doubling compared to placebo treated control animals (Figure 4C). Time to tumor tripling was also significantly increased in estrogen treated mice (data not shown). To confirm that ERβ expression was maintained throughout the course of the experiment, both mRNA and protein levels were determined in residual tumors at the time of sacrifice by RT-PCR and western blotting (Figure 4D and 4E). ERβ protein expression was also monitored by immunohistochemistry in tumor sections (Figure 4F). All of these analyses demonstrated that ERβ expression was maintained and indicated that estrogen treatment may stabilize ERβ protein levels *in vivo*.

### ERβ suppresses a network of genes pertaining to CDK1 and CDK7 function

**Figure 5:**
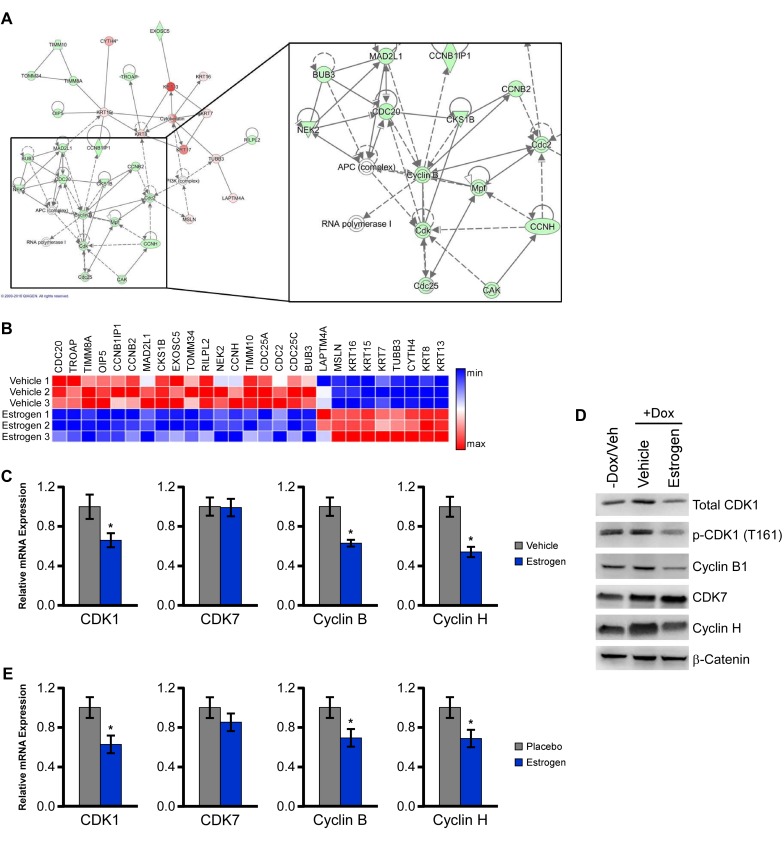
ERβ suppresses a network of genes involved in cell cycle control **A.** Ingenuity pathway analysis was performed using microarray data of E2 regulated genes in MDA-MB-231-ERβ cells following 5 days of treatment and revealed suppression of a cell cycle related network. **B.** Heat map analysis indicating relative expression of genes comprising this network in vehicle and E2 treated MDA-MB-231-ERβ cells. **C.** Independent qPCR analysis of estrogen mediated effects on the expression of CDK1, CDK7, cyclin B1 and cyclin H. **D.** Protein levels of total CDK1, p-CDK1, cyclin B1, CDK7 and cyclin H in Dox-induced estrogen and LY500307 treated MDA-MB-231-ERβ cells relative to non-dox treated controls. B-catenin is shown as a loading control. **E.** mRNA levels of indicated genes in residual tumors isolated from placebo and E2 treated mice. * Denotes significance at the *P* ≤ 0.05 level compared with placebo control.

To determine the mechanisms by which activation of ERβ may elicit its tumor suppressive effects, we interrogated transcriptomic data collected from vehicle and estrogen treated MDA-MB-231-ERβ cells that were generated in our laboratory (data not shown). Ingenuity pathway analysis of estrogen regulated genes identified cell cycle regulation as one of the most significantly regulated networks (Figure 5A). A heat map depicting the estrogen-regulated genes identified within this network is shown in Figure 5B and indicates that the majority of these genes are suppressed in response to estrogen. A number of cyclin-dependent kinases (CDKs) and their respective binding partners were down-regulated including CDK1 (cdc2), cyclin B and cyclin H, while a number of keratins were upregulated. The down-regulation of these targets was confirmed with qPCR analysis (Figure 5C). Decreased protein levels of CDK1, the active form of CDK1 (phospho-CDK1) and cyclin B1 were also observed in response to both estrogen and LY500307 treatment in ERβ expressing MDA-MB-231 cells (Figure 5D). Furthermore, we confirmed that the expression of CDK1, cyclin B, and cyclin H were decreased in the MDA-MB-231-ERβ xenograft tumors isolated from estrogen treated animals relative to placebo treated controls (Figure 5E). These data demonstrate that a number of important cell cycle-related genes are repressed by estrogen and LY500307 treatment in ERβ expressing TNBC cells.

### Inhibition of CDK1 and CDK7 suppresses TNBC cell proliferation

**Figure 6:**
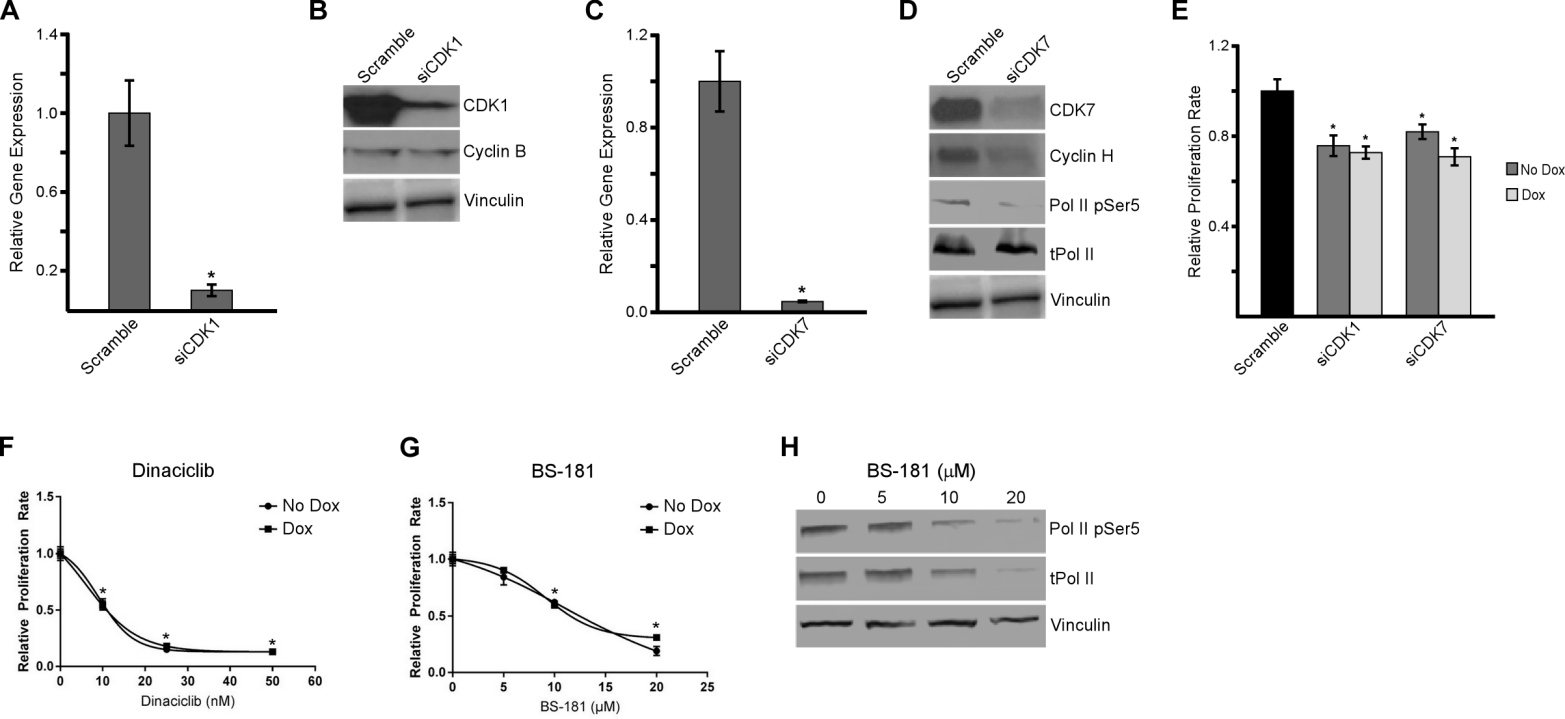
Inhibition of CDK1 and CDK7 decreases proliferation of TNBC cells siRNA-mediated knockdown of CDK1 **A.** and **B.** and CDK7 **C.** and **D.** in MDA-MB-231-ERβ cells were verified at both the mRNA and protein level. The impact of CDK1 (B) and CDK7 (D) knockdown on the protein expression levels of their respective binding partners, cyclin B and cyclin H, as well as RNA Polymerase II phospho-serine 5 for siCDK7 treated cells, are shown. Total RNA Polymerase II and Vinculin are shown as loading controls. **E.** Proliferation rates of MDA-MB-231-ERβ cells six days after siRNA mediated knockdown of CDK1 and CDK7 in the absence (No Dox) and presence (Dox) of ERβ expression relative to scrambled siRNA control transfected cells. Dose response curves indicating the effects of dinaciclib **F.**, a CDK1 inhibitor, and BS-181 **G.**, a CDK7 inhibitor, on the proliferation rates of MDA-MB-231-ERβ cells in the presence and absence of ERβ expression after six days of treatment. The effects of four hours of drug mediated inhibition of CDK7 on the protein levels of total RNA polymerase II and RNA polymerase II phospho-serine 5 were also examined **H.** Vinculin is shown as a loading control. * Denotes significance at the *P* ≤ 0.05 level compared to controls.

Since estrogen and LY500307 resulted in decreased expression of CDK1 and CDK7, we sought to further analyze the roles of these two proteins in regulating cell proliferation and cell cycle progression in TNBC cells. As a first step, we performed siRNA-mediated knockdown of CDK1 and CDK7 in MDA-MB-231 cells. A pool of CDK1 or CDK7 siRNAs was shown to substantially decrease the mRNA (Figure 6A and 6C) and protein levels (Figure 6B and 6D) for each of these genes. Interestingly, knockdown of CDK7 also resulted in decreased expression of its binding partner, cyclin H, as well as the phosphorylation of serine 5 at the C-terminal domain of RNA polymerase II (Figure 6D). siRNA-mediated suppression of CDK1 and CDK7 also resulted in significant decreases in the proliferation rates of MDA-MB-231 cells, effects that were independent of the presence or absence of ERβ expression (Figure 6E). To confirm these effects, dose-response curves were generated using the CDK1 inhibitor, dinaciclib (Figure 6F), and the CDK7 inhibitor, BS-181 (Figure 6G). Proliferation assays revealed potent inhibition of MDA-MB-231 proliferation rates by both drugs in ERβ+ (dox) and ERβ- (no dox) cell lines after six days. As seen with siRNA knockdown, drug inhibition of CDK7 with BS-181 at or above the IC_50_, also showed altered protein levels of total RNA polymerase II and RNA polymerase II phospho-serine 5 (Figure 6H). These data demonstrate that knockdown, or drug-mediated inhibition, of CDK1 and CDK7 results in decreased proliferation in TNBC cells regardless of ERβ expression. In addition, CDK7 inhibition also has an effect on the phosphorylation of RNA polymerase II and therefore might play a dual role in cell cycle progression and transcription.

### Impact of CDK1 and CDK7 inhibition on TNBC cell cycle progression

**Figure 7:**
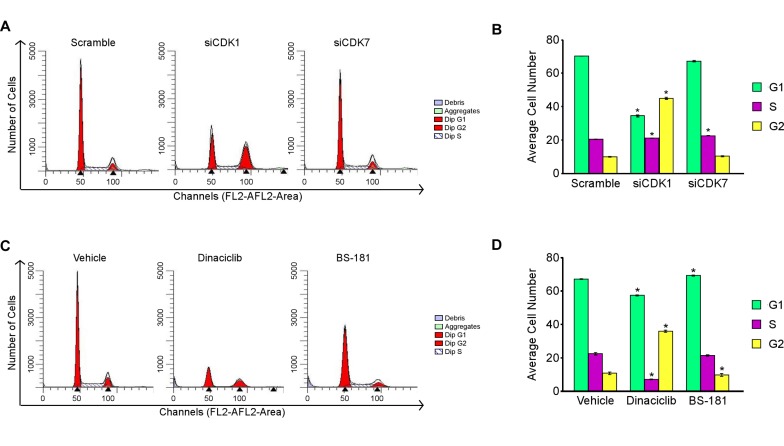
Effects of CDK1 and CDK7 inhibition on cell cycle progression Flow cytometry analysis of MDA-MB-231 cells following 48 hours of siRNA mediated knockdown of CDK1 and CDK7 **A.** and **B.** or drug mediated inhibition of CDK1 (10 nM dinaciclib) and CDK7 (20 µM BS-181) **C.** and **D.** relative to scrambled siRNA control or vehicle treated cells respectively. Transfections were performed with a pool of the two CDK1 or CDK7 siRNAs, or the control siRNA, at a final concentration of 25 nM. * Denotes significance at the *P* ≤ 0.05 level between the indicated treatments and controls.

To further elucidate the roles of CDK1 and CDK7 in modulating TNBC cell proliferation, we next analyzed the effects of siRNA-mediated knockdown of these two genes on cell cycle progression. As shown in Figure 7, knockdown of CDK1 resulted in a dramatic accumulation of MDA-MB-231 cells in the G2-phase (45%) of the cell cycle relative to scrambled siRNA controls (Figure 7A and 7B). Interestingly, knockdown of CDK7 did not have a significant effect on cell cycle progression (Figure 7A and 7B) in spite of the fact that cell proliferation rates were decreased under these same conditions (Figure 6E). This effect could in part be due to CDK7’s role in transcription where it is known to phosphorylate the C-terminal domain of RNA polymerase II at Serine 5 [25]. Drug mediated inhibition of CDK1 with dinaciclib also resulted in a G2 arrest (Figure 7C and 7D) similar to that of the siRNA. Blockade of CDK7 with BS-181 had no significant effect on cell cycle progression (Figure 7C and 7D) as was also shown to be the case in CDK7 siRNA transfected cells. Overall these data demonstrate that suppression of CDK1 results in a G2 phase cell cycle arrest while blockade of CDK7 function has no impact on cell cycle progression of TNBC cells.

## DISCUSSION

ERβ is a tumor suppressor whose expression is associated with a better prognosis in breast cancer [6, 26-35]. As ERβ is expressed in approximately 30% of TNBCs we sought to determine the therapeutic potential of targeting ERβ in TNBC. In this manuscript, we demonstrate that ligand-mediated activation of ERβ with estrogen, or the ERβ selective agonist LY500307, resulted in anti-proliferative effects *in vitro* and suppression of tumor progression *in vivo.* Activation of ERβ was shown to induce cell cycle arrest, but not apoptosis. ERβ was also shown to inhibit the expression of a number of cell cycle-related genes both *in vitro* and *in vivo* including CDK1, cyclin B, and cyclin H. siRNA and drug mediated inhibition of CDK1 resulted in decreased proliferation of MDA-MB-231 cells due to a G2/M phase cell cycle arrest, effects that were independent of ERβ expression. Inhibition of CDK7 using siRNA and BS-181 resulted in decreased proliferation of TNBC cells, but had no effect on cell cycle progression.

In this manuscript, we report for the first time that low concentrations of LY500307, a potent ERβ selective agonist, can not only activate ERβ but also suppress proliferation of ERβ expressing TNBC cells. These anti-proliferative effects are an extension of previous studies by our laboratory and others demonstrating that ligand-mediated activation of ERβ results in anti-proliferative effects in multiple cancer cell lines [6-8, 36]. In addition, we showed that estrogen treatment of mice harboring ERβ+ MDA-MB-231 xenografts results in significant inhibition of tumor growth, and in some animals induced complete tumor regression. These findings are in agreement with a previous collaborative study with Dr. Wei Xu’s group in which estrogen treatment induced anti-tumor effects in ERβ expressing MDA-MB-468 xenografts [8].

Previous findings from our laboratory have demonstrated that estrogen treatment of ERβ expressing Hs578T TNBC cells inhibits proliferation primarily by inducing a G1 cell cycle arrest [7]. We have confirmed these observations in the present study using MDA-MB-231-ERβ cells. In the MDA-MB-231-ERβ model, we also observed a slight, but insignificant, increase in G2 arrest. These findings agree with previous studies that have linked ligand-mediated activation of ERβ to both G1 and G2 cell cycle arrest in multiple types of cancer cell lines [10, 11, 37-40]. In addition to cell cycle analysis, we performed an Annexin V/PI assay to determine if programmed-cell death contributed to the observed decreased rates of proliferation. Interestingly, when ERβ is expressed, the basal number of cells undergoing apoptosis decreased compared to when ERβ is absent. These effects of ERβ on decreased rates of apoptosis have been observed previously and may be related to the decreased rates of cell growth [41]. These data indicated that alterations in apoptosis do not contribute to the tumor suppressive effects of ERβ in this model system and instead that the majority of this phenotype is driven by increases in cell cycle arrest.

To understand the potential mechanisms of action by which ERβ suppresses cell cycle progression, we performed ingenuity pathway analysis on existing gene expression studies conducted by our laboratory. In this analysis, one of the most significantly altered biological pathways following estrogen treatment of MDA-MB-231-ERβ cells was a network of genes involved in cell cycle regulation. Among these genes were CDK1, cyclin B and cyclin H which were all significantly suppressed by estrogen treatment. Unliganded ERβ has previously been shown to repress the expression of cyclin B1, which in turn decreases the activity of CDK1 and results in a G2 cell cycle arrest in MCF7 cells [10]. Another interesting observation was that a number of keratins were also upregulated. Keratins are known markers of differentiation that can be associated with histological grade, however, no observations of differential tumor histology were observed in the animal xenografts [42, 43]. To the best of our knowledge, our findings are the first to report that ERβ suppresses the functions of CDK1 and CDK7 (through suppression of cyclin H) in TNBC.

Since CDK1 is required for mitosis, approaches to target the CDK1-cyclin B complex are appealing for cancer therapy. In fact, agents targeting CDK1 or cyclin B have been shown to effectively block tumor growth and progression of multiple forms of cancer [44-48]. While a pure CDK1 inhibitor does not yet exist, a number of pan-CDK inhibitors, including dinaciclib, have been developed [49, 50]. Dinacilib targets CDK1, CDK2, CDK5 and CDK9 with *in vitro* IC_50_ values of 3 nM 1 nM, 1 nM and 4 nM, respectively. At the concentrations utilized in this study, dinaciclib would have an effect on all four of these CDKs. Our results demonstrated a potent G2/M cell cycle arrest indicative of CDK1 inhibition in TNBC cells and significantly decreased proliferation rate. These findings correlate well with other pre-clinical studies demonstrating inhibition of cell proliferation by siRNA mediated suppression or dinaciclib treatment in other types of cancer cell lines [22, 50-53]. Furthermore, clinical studies of dinaciclib have demonstrated efficacy in both solid tumors and relapsed multiple myeloma with a manageable safety profile [54, 55].

In addition to CDK1, we also demonstrated that CDK7 function is likely to be repressed given the decreased expression of cyclin H following estrogen treatment of ERβ expressing MDA-MB-231 cells and xenograft tumors. CDK7 knockdown was shown to decrease cyclin H protein levels, an effect that has also been observed in a previous report [56]. CDK7, unlike other CDKs, plays a dual role in regulating both cell cycle progression and transcription [57, 58]. In regard to cell cycle progression, inhibition of CDK7, specifically during the G2-phase of the cell cycle, prevents entry into mitosis by disrupting the assembly and phosphorylation of the CDK1-cyclin B complex [59]. On the other hand, CDK7 plays an important role in transcription where it phosphorylates serine 5 at the C-terminal domain of RNA polymerase II [58]. Here we demonstrate that knockdown or drug inhibition of CDK7 did indeed have an effect on the phosphorylation of RNA polymerase II at serine 5. In the present report, inhibition of CDK7 resulted in decreased proliferation of TNBC cells, which could be due to the transcriptional activity of CDK7 rather than its influence on cell cycle progression as no effect was observed. These findings are in agreement with a previous study demonstrating that some TNBC cells are “addicted” to CDK7 mediated transcription, and that suppression of CDK7 function elicits tumor suppressive effects [59].

In conclusion, these data indicate that ligand-mediated activation of ERβ in TNBC cells results in decreased proliferation rates, cell cycle arrest and suppression of tumor growth. These effects are likely mediated in part by suppression of CDK1 and CDK7 function. We provide further evidence that inhibition of CDK1 and CDK7 expression/function also results in decreased proliferation of TNBC cells, effects that are independent of ERβ expression or estrogen treatment. These findings support the notion that CDK1 and CDK7 serve as clinically relevant therapeutic targets in TNBC, an area of study that has yet to be fully explored.

## MATERIALS AND METHODS

### Cell culture and chemicals

Doxycycline (dox)-inducible MDA-MB-231-ERβ cells were established in our laboratory as previously described [6, 7] and were maintained in phenol red-free DMEM/F12 medium supplemented with 10% fetal bovine serum (FBS), 1% antibiotic-antimycotic (AA), 5 mg/L blasticidin S and 500 mg/L zeocin and cultured in a humidified 37°C incubator with 5% CO_2_. HyClone™ charcoal/dextran stripped FBS (CS-FBS) was purchased from GE Healthcare Life Sciences (Pittsburgh, PA). For xenograft models, the MDA-MB-231-ERβ-Luc cell line was generated by stably integrating the pLNCX-FLuc (firefly luciferase) vector into the doxycycline-inducible ERβ1-expressing MDA-MB-231 cell line. MDA-MB-231-ERβ-Luc cells were maintained in identical medium with the addition of 500 µg/L puromycin. 17β-estradiol (E2) and doxycycline (Dox) were purchased from Sigma-Aldrich (St. Louis, MO). The ERβ-selective agonist, LY500307, was obtained from Eli Lilly (Indianapolis, IN). Dinaciclib (SCH727965) and BS-181 HCl were purchased from Selleckchem (Houston, TX).

### Transient transfection and luciferase assay analysis

MDA-MB-231-ERβ cells were plated at 35,000 cells/well in 24-well plates with 10% CS-FBS containing media supplemented with 100 ng/ml dox for 24 hours. Cells were transfected with 100 ng of the estrogen response element (ERE) luciferase reporter construct using FuGENE 6 transfection reagent (Roche) in CS-FBS containing media. The next day, cells were washed twice with 1X PBS and treated with CS-FBS containing media supplemented with 100 ng/ml Dox and ethanol control, 1 nM estrogen, 1, 10, 100, and 1000 nM LY500307 for 24 hours. Cells were harvested using 1X Passive Lysis Buffer (Promega, Madison, WI) and equal amounts of protein extract were assayed using luciferase assay reagent and a Glomax-Dual Luminometer (Promega).

### Proliferation assays

Relative proliferation rates were determined using crystal violet assays. Briefly, cells were plated in replicates of eight at a density of 2,000 cells per well in 96-well plates using 10% CS-FBS containing phenol-red free medium in the presence (100 ng/mL) or absence of Dox as indicated. Twenty-four hours later, cells were treated with vehicle (ethanol), 1 nM E2, or various concentrations of LY500307, dinaciclib, BS-181 or siRNAs as indicated. After six days of treatment, cells were fixed with glutaraldehyde and stained with crystal violet. Staining was solubilized with 100 nM sodium citrate and quantified using a plate reader at wavelength 550 nm. Replicates were averaged among treatment groups and values were normalized to vehicle control treated cells. Experiments were repeated a minimum of three times and a representative data set is shown. Student *t*-tests were performed to determine significance between treatments and vehicle controls.

### Flow cytometry

MDA-MB-231-ERβ cells were plated in 10 cm tissue culture dishes in 10% CS-FBS containing medium. Following 24 hours of treatment in the absence or presence of Dox, cells were treated with ERβ ligands, drugs or siRNAs. ERβ ligand treatment was performed for both 24 hours and 5 days while drug inhibitors or siRNA knockdowns were harvested and assayed after 24 hours. Propidium iodide (PI) (Sigma-Aldrich) staining was performed for cell cycle analysis as previously described [60] and AnnexinV/PI (BD Biosciences, Franklin Lakes, NJ) staining for apoptosis as previously described [61]. Briefly, on the day of harvest, cells were washed once with 1X PBS and dissociated using 1 mL TripLE™ (Invitrogen). For cell cycle analysis, cells were fixed, permeabilized, and incubated with RNAse A solution (0.1 mg/mL in 0.1% (w/w) sodium citrate, Roche, Indianapolis, IN) for 15 minutes. PI (0.1% mg/mL in 0.1% sodium citrate) was added and cells were incubated at room temperature in the dark for 15 minutes prior to analysis by flow cytometry. For apoptosis analysis, following dissociation, cells were stained with Annexin V on ice in the dark for 30 minutes. Propidium iodide was then added and samples were analyzed within 1 hour. Flow cytometry was performed using the FACSCalibur flow machine in the Mayo Clinic Flow Cytometry Core Facility (Rochester, MN). ModFit LT software was utilized to determine the percentage of cells in G1, S, and G2/M phases for each treatment and subsequently averaged across triplicate experiments. CellQuest Pro software was implemented to determine the percent Annexin V-positive and Annexin V/PI-positive cells for each treatment which were considered to be apoptotic. Values were summed for each sample and averaged among treatment groups.

### Apoptosis protein array

MDA-MB-231-ERβ cells were plated in 10 cm tissue culture dishes in the presence of Dox and treated with ethanol vehicle or 1 nM estrogen for 5 days in 10% CS-FBS containing medium. A human apoptosis array kit was purchased from R&D systems, Inc. (Minneapolis, MN) and the assay was performed following the manufacturer’s protocol. Briefly, nitrocellulose membranes containing capture and control antibodies spotted in duplicate for each target were blocked for an hour at room temperature. Four hundred µg of each cell lysate was applied to respective membranes and incubated overnight at 4°C. The next day, membranes were washed and incubated with a cocktail of biotinylated primary detection antibodies for an hour at room temperature. After washing, membranes were incubated with Streptavidin-HRP for 30 minutes. One mL of Chemi Reagent Mix was applied to each membrane for 1 minute before removal and exposure to x-ray film. Quantification was performed using ImageJ software.

### MDA-MB-231-ERβ xenograft studies

MDA-MB-231-ERβ-Luc cells were grown at 37°C with 5% CO_2_ until approximately 80% confluency at which time they were trypsinized, counted and resuspended at 1.0 x 10^6^ cells/100 µL in equal volumes of 1X PBS and matrigel. Cells were injected into the right flank of six to eight week old ovariectomized female nude mice purchased from the Jackson Laboratory (Bar Harbor, ME). Tumor volumes were measured weekly using digital calipers. Once the average tumor volume for all animals reached approximately 100 mm^3^, mouse diets were changed to 200 mg/kg Dox-containing chow (TD.98186, Envigo Tekland Diet, Madison, WI) to induce tumoral expression of ERβ and animals were randomized to placebo or 17β-estradiol (0.54 mg/90-day release) pellets (*n* = 8 animals per group). Pellets were purchased from Innovative Research of America (Sarasota, FL) and were implanted into the nape of the neck using a trochar. Tumor volumes were monitored weekly with digital calipers using the formula: *Tumor volume* = ½(*length* × *width*^*2*^*)*. Additionally, luciferase-based imaging was performed as previously described [62] using the Xenogen IVIS 200 Imaging System. In brief, mice were sedated with Isoflurane and 1 mg D-luciferin (10 mg/mL in PBS) was administered *via* intraperitoneal injection. Images were captured approximately 15 minutes after injection to allow for the development of complete luciferase activity. All animal work was carried out in strict accordance with the recommendations in the Guide for the Care and Use of Laboratory Animals of the National Institutes of Health. The protocol was approved by the Mayo Clinic Institutional Animal Care and Use Committee (Permit Number: A33015).

At the time of sacrifice, tumor xenografts were dissected and an approximately 5 mm thick section was obtained through the center of each tumor and was processed for formalin fixation and paraffin embedding. Five µm sections were cut and utilized for ERβ IHC analysis. Additional tumor pieces were processed for RNA extraction using TRIzol^®^ Reagent (ThermoFisher Scientific, Waltham, MA) and protein extraction using RIPA buffer as described below.

### Immunohistochemistry

Five micron formalin-fixed, paraffin embedded sections were cut for immunostaining and analyzed as previously described [63]. IHC staining was performed at the Pathology Research Core (Mayo Clinic, Rochester, MN) using the Leica Bond RX stainer (Leica). Briefly, tissue slides were dewaxed and retrieved on-line using the following reagents Bond Dewax (Leica) and Epitope Retrieval 2, EDTA based (Leica Biosystems Inc. Buffalo Grove, IL). Tissue slides were retrieved for 30 minutes. The ERβ1 PPG5/10 antibody (Thermo Scientific, Waltham, MA) was diluted 1:75 in Background Reducing Antibody Diluent (Dako, Agilent Technologies, Santa Clara, CA) and incubated for 30 minutes. This antibody has been shown to be highly specific and sensitive for detection of only the full-length form of this receptor in previous IHC studies [59-61].

### Biological pathway analysis

Existing microarray data from our laboratory was analyzed for pathways that were significantly altered following 1 nM E2 treatment for five days using the Ingenuity Pathway Analysis software (IPA, Ingenuity Systems, Inc., Redwood City, CA; http://www.ingenuity.com). Significant canonical pathways in which the Differentially Expressed Genes (DEGs) in the tested samples were enriched were identified. The IPA program applies Fisher’s exact test to calculate a p-value that represents the probability of the DEGs in the pathway being found together due to random chance. Specifically, genes identified in the microarray with differential expression p-values ≤0.05 and fold-changes ≥1.5 were used as focus genes. Pathways with *p*-values < 0.05 were significantly enriched.

### Real-time RT-PCR

One microgram of total RNA was reverse transcribed using the iScript™ cDNA Synthesis Kit (Bio-Rad). Real-time PCR was performed in triplicate using a Bio-Rad CFX Real-Time PCR Detection system (Hercules, CA). The PerfeCTa™ SYBR Green Fast Mix™ (Quanta Biosciences, Gaithersburg, MD) PCR kit was used according to the manufacturer’s instructions and the following cycling conditions were employed: 95°C for 2 minutes followed by 40 cycles of 95°C for 5 seconds and 60°C for 30 seconds. Melt curves were generated to ensure amplification of a single PCR product. Quantitation of the PCR results were calculated based on the threshold cycle (C_t_) and normalized to the controls, TATA Binding Protein (TBP) or 18S ribosomal RNA. All PCR primers were designed using Primer3 software (http://bioinfo.ut.ee/primer3-0.4.0/primer3/) and were purchased from Integrated DNA Technologies (Coralville, IA). Primer sequences are listed in Table 1.

**Table 1:** Primer sets used in qRT-PCR assays

	Primer Sequence (5'-3')	
Gene	Forward	Reverse
TBP	AGTTGTACAGAAGTTGGGTTTTC	AACAATTCTGGGTTTGATCATTC
18S	AGCCTGAGAAACGGCTACCA	GGGTCGGGAGTGGGTAATTT
ERβ	GATAAAAACCGGCGCAAGAG	TCACCATTCCCACTTCGTAACA
CDK1	GGGCACTCCCAATAATGAAGTG	AGGCTTCCTGGTTTCCATTTG
Cyclin B1	TGGCCAAATACCTGATGGAACT	GCTGCAATTTGAGAAGGAGGAA
Cyclin H	TGTTGTGGGTACGGCTTGTA	AAGTGCCTTCTCCTGTCCAA
CDK7	GGTCTCCTTGATGCTTTTGG	GGCTTTGATGTGTGATGGTG

### Western blot analysis

Total protein lysates were prepared using RIPA buffer (25 mM Tris pH 7.4, 150 nM NaCl, 1% sodium deoxycholate, 1% NP40, 0.1% SDS). RIPA buffer was supplemented with an EDTA-free protease inhibitor cocktail and phosphatase inhibitor cocktail (Roche, Indianapolis, IN) prior to use. Protein concentrations were determined using a Bradford assay and equal amounts of lysate were loaded onto 4-15% gradient SDS-PAGE gels, transferred to PVDF (Bio-Rad, Hercules, CA) and blocked with 5% milk in TBST for one hour at room temperature. Blots were probed with the following primary antibodies: CDK7 (#2916), cyclin B1 (#4138), cyclin H (#2927), phospho-CDK1 Thr161 (#9114), and total CDK1 (#9116) from Cell Signaling Technology (Danvers, MA), β-catenin (#05-665) and Total RNA-Polymerase II (#05-623) from Millipore (Billerica, MA), Vinculin (ab129002) and RNA Polymerase II phospho-Ser 5 (ab5131) from Abcam (Cambridge, MA). Primary antibodies were prepared in either 5% milk or 5% BSA in TBST per company recommendations and incubated overnight at 4°C. After washing, blots were incubated with anti-mouse sc-2005 and anti-rabbit sc-2004 secondary antibodies from Santa Cruz Biotechnology (Dallas, TX) for one hour at room temperature. Blots were imaged on the Odyssey Fc (LI-COR, Lincoln, NE) system with the imager set to capture the 700 nm and chemi channels for 30 seconds and 10 minutes, respectively.

For infrared fluorescence, protein lysates were prepared, loaded, and the gel was run exactly as described above. Proteins were transferred onto FL PVDF membranes (Sigma, St. Louis, MO) and blocked with Odyssey blocking buffer for one hour at room temperature. Blots were probed with the following primary antibodies: ERβ (#8974) from Santa Cruz (Dallas, TX) and B-actin (#A2228) from Sigma (St. Louis, MO). Primary antibodies were prepared in Odyssey PBS buffer and incubated overnight at 4°C. After washing, blots were incubated with anti-mouse 680RD or anti-rabbit 800CW secondary antibodies for one hour at room temperature protected from the light. Blots were imaged on the Odyssey Fc (LI-COR, Lincoln, NE) system with the imager set to capture the 700 nm and 800 nm channels.

### CDK1 and CDK7 inhibition

For knockdown experiments, two pre-designed siRNAs targeting both CDK1 and CDK7 were purchased from Dharmacon (Lafayette, CO) and consisted of the following sequences: CDK1-9: 5’-GUACAGAUCUCCAGAAGUA-3’, CDK1-10: 5’-GAUCAACUCUUCAGGAUUU-3’, CDK7-5: 5’-GGACAUAGAUCAGAAGCUA-3’, CDK7-6: 5’-CAAUAGAGCUUAUACACAU-3’. The siGENOME Non-Targeting siRNA Pool 1 (Dharmacon; D-001206-13) was used as a negative control. Transfections were performed with a pool of the two CDK1 or CDK7 siRNAs, or the control siRNA, using Dharmafect Reagent 1 (Dharmacon; T-2001) according to the manufacturer’s instructions. A final concentration of 25 nM of total siRNA was used for all experiments.

Drug mediated inhibition of CDK1 and CDK7 was performed using dinaciclib (CDK1) and BS-181 (CDK7). Dose-response proliferation assays were performed for six days to determine the inhibitory concentration of 50% of our MDA-MB-231 cells (IC_50_) for each CDK inhibitor. The CDK inhibitors were tested at the following concentrations: dinaciclib at 10 nM, 25 nM and 50 nM and BS-181 at 5 µM, 10 µM, and 20 µM. Proliferation assays were performed after six days while cell cycle analysis *via* flow cytometry was performed after 48 hours following knockdown or drug inhibition of CDK1 and CKD7 as described above.

### Statistical analysis

All of the *in vitro* experiments were conducted with a minimum of three biological replicates with 3-6 technical replicates per assay and representative data sets are shown. A students *t*-test was used to determine statistical significance between treatments relative to controls. P-values ≤ 0.05 were considered to be statistically significant. For the animal models, 8 mice per treatment group were utilized and a log-rank test was used to assess whether time to tumor doubling differed between treatment groups. All graphs and analyses were processed using SAS 9.4.

## Abbreviations

TNBC: triple negative breast cancer
ERβ: estrogen receptor beta
ERα: estrogen receptor alpha
PR: progesterone receptor
HER2: human epidermal growth factor receptor 2
CDK: cyclin-dependent kinase
Dox: doxycycline
CS-FBS: charcoal-stripped fetal bovine serum
E2: 17β-estradiol
DFS: disease-free survival
OS: overall survival
